# Delayed-onset contact dermatitis caused by olanexidine gluconate antiseptic solution: effect of wiping off the remaining drug solution in two cases

**DOI:** 10.1186/s40981-023-00604-0

**Published:** 2023-03-08

**Authors:** Kasumi Boki Yamamoto, Keisuke Fujii, Kazuhiro Mizumoto, Tadashi Tanioku, Tomoyuki Kawamata

**Affiliations:** Department of Anesthesiology, School of Medicine, Wakayama Medical University Hospital, 811-1 Kimiidera, Wakayama, 641-8510 Japan

**Keywords:** Disinfection, Dermatitis, Olanexidine

## Abstract

**Background:**

Olanexidine glucuronide (Olanedine®), an antiseptic solution may cause skin dermatitis around one week after disinfection. Although removal after the procedure is recommended to avoid skin dermatitis, whether it is effective for preventing skin dermatitis has not been documented in detail in the literature.

**Case presentation:**

We encountered two cases of delayed-onset contact dermatitis by Olanedine®. In both cases, the patient’s back was disinfected with Olanedine® and was covered with a surgical drape for epidural catheterization. After catheterization and removal of the surgical drape, the insertion site of the catheter was covered with a film dressing, then the epidural catheter was taped to the back. On the third postoperative day, the epidural catheter was removed. On the seventh postoperative day, the patients reported pruritus on the back, where an erythematous papule rash was observed. However, it was not observed at the site covered by the tape to secure the epidural catheter or by the tape of the surgical drape. Symptoms were relieved with oral or topical steroids by the time of discharge.

**Conclusion:**

Wiping off the remaining Olanedine® even a few days after disinfection may be helpful not only for reducing symptoms but also for preventing the development of contact dermatitis.

## Background

Olanexidine gluconate (Olanedine® Antiseptic Solution 1.5%, Otsuka Pharmaceutical Factory, Tokushima, Japan) is a biguanide disinfectant with broad-spectrum, with more potent bactericidal activity against methicillin-resistant *Staphylococcus aureus* and vancomycin-resistant enterococci than chlorhexidine [1, 2]. However, delayed skin dermatitis may appear about 1 week after disinfection [3, 4]. The manufacturer recommends the removal of Olanedine® after the procedure, although the effectiveness of removal on the skin dermatitis has not been documented in the literature. Here, we report two cases in which wiping off the remaining drug solution even 3 days after disinfection was considered to be effective in reducing symptoms.

## Case presentation

### Case 1

**Fig. 1:**
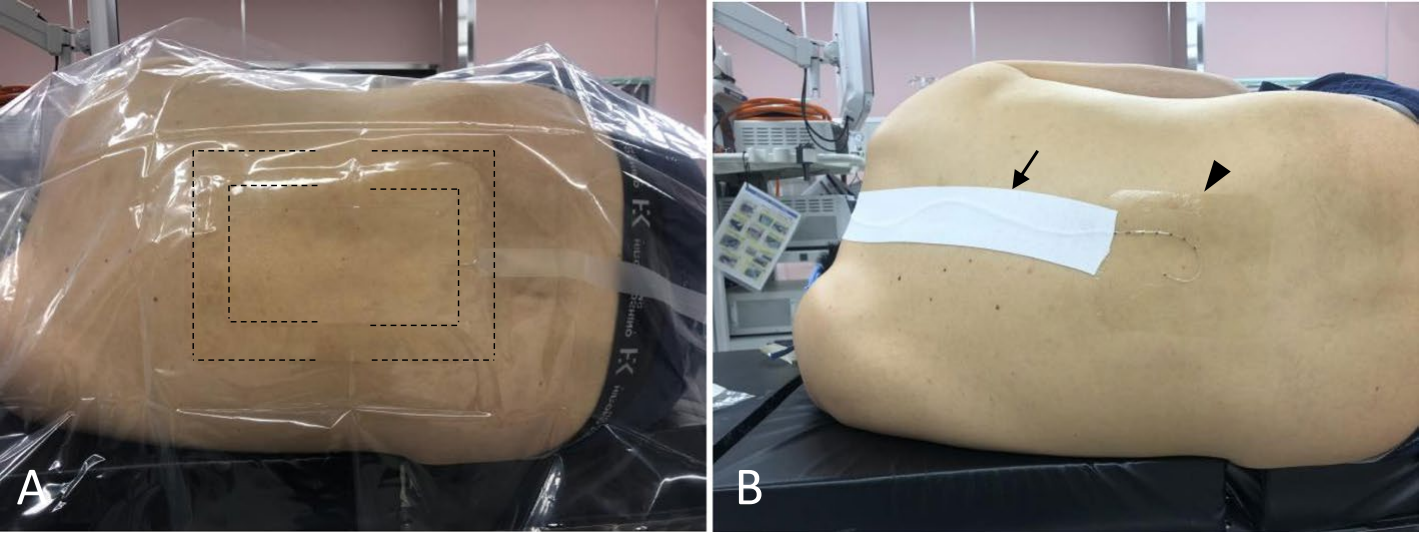
Preparation of epidural tubing in our hospital. In **A**, the black dotted lines and black arrows indicate the tape of the surgical drape. In **B**, the black arrow and arrowhead indicate the tape used to secure the epidural catheter and a film dressing, respectively

**Fig. 2:**
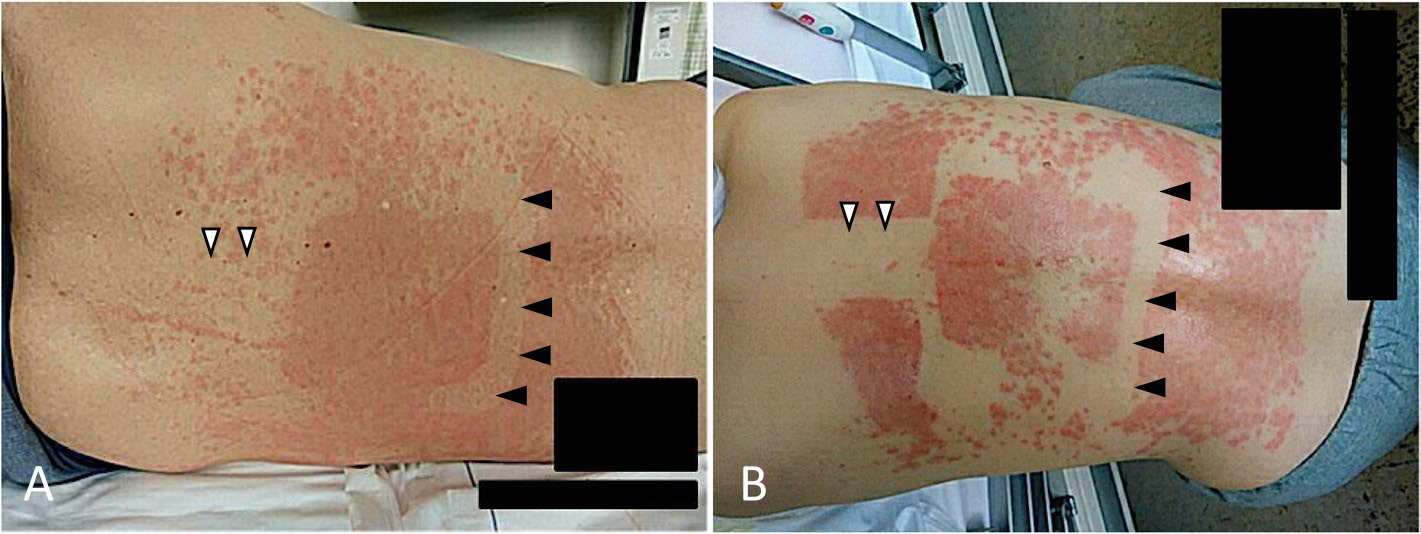
Erythematous papules on the back after disinfection with Olanedine Antiseptic Solution 1.5% (Olanedine®). **A** and **B** show erythematous papules observed in the area disinfected with Olanedine® in cases 1 and 2, respectively. An erythematous papule rash was not observed in the area where the surgical drape was taped (black arrowheads) or the area where the patient’s back was taped to secure an epidural catheter (white arrowheads) in either case

A 69-year-old man was scheduled to undergo a robotic-assisted pyloric gastrectomy for gastric cancer. He had a history of smoking and chronic obstructive pulmonary disease, but he had no history of allergies. In the operating room, his back was disinfected with Olanedine® and was covered with a surgical drape before epidural catheterization (Fig. 1A). After catheterization, the surgical drape was removed, and his back was not wiped off with saline or something. Then, the site of insertion of an epidural catheter was covered with a transparent film dressing (3M™ Tegaderm™ Transparent Film Dressing, 3M Japan, Tokyo), and an epidural catheter was taped to his back (Fig. 1B). His abdomen was also disinfected with Olanedine® before surgery. The surgery was completed without problems. After surgery, his abdomen was wiped off with normal saline. On the third postoperative day, the epidural catheter was removed. On the seventh postoperative day, the patient reported an itchy sensation on his back, and erythematous papules were observed in the disinfected area on the back on the eighth postoperative day. An erythematous papule rash was not observed at the site of the tape of the surgical drape for epidural catheterization, at the site of the tape used to secure the epidural catheter on his back (Fig. 2A) or abdomen. Delayed erythematous papules and itching were diagnosed by our dermatologists as contact dermatitis due to Olanedine® from his symptoms and clinical course. Treatment with oral olopatadine hydrochloride, a histamine H1 receptor antagonist, was initiated and his symptoms were relieved by the time of discharge.

### Case 2

A 62-year-old woman was scheduled to undergo laparoscopy-assisted pyloric gastrectomy for gastric cancer. She had no history of allergies. In the operating room, her back was prepared as in case 1 for epidural catheterization. Her abdomen was also disinfected with Olanedine® before surgery, and the surgery was completed without problems. After surgery, her abdomen was wiped with normal saline. On the third postoperative day, the epidural catheter was removed. As in case 1, on the seventh postoperative day, she reported itching on her back, and erythematous papules were observed in the disinfected area on her back. Erythematous papules were not observed at the tape site of the surgical drape for epidural catheterization, at the site of the tape used to secure the epidural catheter on her back (Fig. 2B) or on her abdomen. Our dermatologists diagnosed delayed-contact dermatitis due to Olanedine® from her symptoms and clinical course. Treatment with topical betamethasone and oral fexofenadine hydrochloride, a histamine H1 receptor antagonist, was initiated. The itching and erythematous papules were decreased on the 9th postoperative day. Symptoms were relieved by the time of discharge.

## Discussion

We described two cases of delayed-onset contact dermatitis caused by Olanedine®. Olanedine®-induced itching, erythematous papule rash, or dermatitis has been reported to occur in 1–2% of cases [5]. Olanedine®-induced allergic reaction is considered to be caused by olanexidine gluconate [4]. Olanedine® includes polyoxyethylene (20) polyoxypropylene (20) glycol, glucono-δ-lactone, and sodium hydroxide as the additives in addition to olanexidine gluconate, and the additives may therefore be involved in delayed dermatitis. Recently, 2-day closed patch tests using filter paper with the test solution that had been dried before application have been recommended in order to correctly diagnose antiseptic-induced allergic contact dermatitis [6]. Drug residues on the skin are suspected as a cause of delayed contact dermatitis. Areas in which the tape of the surgical drape and the epidural catheter fixation tape had been tightly adhered were not symptomatic in our cases. On the other hand, symptoms in areas in which a film dressing was loosely adhered, such as the site of insertion of an epidural catheter, were similar to those in areas without the tape of the surgical drape and the epidural catheter fixation tape. We assume that Olanedine® stuck to the drape and the fixation tape and was consequently wiped off. In both cases, while the drape was quickly removed after the procedure, the epidural catheter fixation tape was removed 3 days after the procedure. This evidence suggests that wiping off the remaining drug solution is effective for reducing symptoms and preventing the development of contact dermatitis even 3 days after the procedure. However, the possibility that the adhesive fabric taps used to secure the epidural catheter absorbed Olanedine® and considerably reduced the concentration of Olanedine® on the skin under the tape should also be considered.

After we experienced two delayed-contact dermatitis after disinfection of Olanedine®, removal of Olanedine® after the procedure was recommended by the manufacturer. Now, we routinely wipe off Olanedine® with saline gauze after the procedure.

## Conclusion

Two patients had delayed-onset contact dermatitis caused by Olanedine®. Wiping off Olanedine® even a few days after the procedure may be helpful not only for reducing symptoms of contact dermatitis including erythematous papules and itching sensation but also for preventing the development of contact dermatitis.

## Data Availability

Not applicable.

## Abbreviation

OAS: Olanedine antiseptic solution 1.5%
